# Adolescents’ credibility justifications when evaluating online texts

**DOI:** 10.1007/s10639-022-10907-x

**Published:** 2022-02-10

**Authors:** Carita Kiili, Ivar Bråten, Helge I. Strømsø, Michelle Schira Hagerman, Eija Räikkönen, Anne Jyrkiäinen

**Affiliations:** 1 Faculty of Education and Culture, Tampere University, P.O. Box 700, 33014 Tampere, Finland; 2 Department of Education, University of Oslo, P.O. Box 1092 Blindern, 0137 Oslo, Norway; 3 Faculty of Education, University of Ottawa, 145 Jean-Jacques-Lussier Private, Ottawa, ON K1N 6N5 Canada; 4 Faculty of Education and Psychology, University of Jyväskylä, P.O. Box 35, Jyväskylä, 40014 Finland

**Keywords:** Sourcing, Credibility justification, Online evaluation, Adolescents, Epistemic beliefs, Internet-specific epistemic justification beliefs, Behavioral engagement

## Abstract

Research has shown that students differ in their abilities to evaluate the credibility of online texts, and, in general, many perform poorly on online evaluation tasks. This study extended current knowledge by examining students’ abilities to justify the credibility of online texts from different perspectives, thus providing a more nuanced understanding of students’ credibility evaluation ability. We examined how upper secondary school students (N = 73; aged 16 to 17) evaluated author expertise, author intention, the publication venue, and the quality of evidence when reading four texts about the effects of sugar consumption in a web-based environment. Additionally, we examined how students’ prior topic knowledge, Internet-specific justification beliefs, and time on task were associated with their credibility justifications. Students evaluated author expertise, author intention, the venue, and the quality of evidence for each text on a six-point scale and provided written justifications for their evaluations. While students’ credibility evaluations were quite accurate, their credibility justifications lacked sophistication. Inter-individual differences were considerable, however. Regression analysis revealed that time on task was a statistically significant unique predictor of students’ credibility justifications. Instructional implications are discussed.

The Internet has democratized access to and publishing of information. Consequently, the responsibility for evaluating information has increasingly been put on readers’ shoulders (Thomm & Bromme, 2012). Therefore, readers need to be equipped with skills that enable them to make judgments about who and what to believe online. To be able to answer these questions, readers are required to pay attention to the source credibility and plausibility of claims and evidence (Sinatra & Lombardi, 2020). To develop as critical readers, they also need to consider an additional question: “How do I know?” (Hofer, 2016). Although several studies have investigated adolescent readers’ abilities to evaluate the credibility of information in offline and online contexts, these studies have focused on the trustworthiness of the source and topic relevance (Macedo-Rouet et al., 2019), author’s expertise and point of view (Coiro et al., 2015; Forzani, 2018), or credibility evaluation in general (Barzilai & Zohar, 2012; Kiili et al., 2019). There is a need, therefore, for studies that systematically examine adolescents’ capacity to evaluate both the trustworthiness of diverse information sources and the quality of the evidence they present.

In this study, we addressed how upper secondary school students evaluate the credibility of online texts in terms of four central aspects of credibility: author expertise, author intention, publication practices of the venue, and quality of evidence. In addition, we examined how students’ prior topic knowledge, Internet-specific epistemic justification beliefs, and behavioral engagement were associated with students’ credibility justifications. By doing this, we uniquely extended prior research in the area by addressing adolescents’ credibility evaluations more broadly in combination with individual differences that may predict their justifications for those evaluations.

## Evaluation of Online Texts

On the Internet, readers are often confronted with contradictory claims and evidence on various topics, such as environmental and health issues (Barzilai & Chinn, 2020; Freeman et al., 2020). The ease of publishing on the Internet implies that authors with varying degrees of expertise and a range of intentions, including persuasion and even deception, can find a wide readership. To make judgments about the author’s trustworthiness, readers can pay attention to several source features, including indicators of the author’s expertise and benevolence (Hendriks et al., 2016; Stadtler & Bromme, 2014). Thus, to decide whether the author is knowledgeable in the domain of the text, readers can look for information about the authors’ education, credentials, experience, and affiliation. Readers should be aware that when authors write about topics for which they have insufficient knowledge, they risk conveying inaccurate information (Afflerbach et al., 2014). Readers can also make inferences about the author’s benevolent intentions, that is, his or her good will towards others or society (Hendriks et al., 2015). They can look for indicators of integrity that would show good character, values that align with a commitment to public service, and whether the author acts in good faith without pursuing personal benefits (Hendriks et al., 2016). Readers are also challenged by varied commitments to editorial or quality control across publication venues. Considering the reputation of the website and acknowledging editorial processes or guidelines can offer additional information for making credibility evaluations.

However, evaluation of source information may not be sufficient to make adequate credibility judgments. Readers should also be equipped to evaluate the plausibility of competing arguments introduced by the sources (Sinatra & Lombardi, 2020). Nussbaum (2020) has developed a set of critical questions that can assist students in evaluating arguments. These questions guide students first to identify the claim and supporting evidence. Identification of the argument structure is the prerequisite for assessing the correctness and coherence of reasoning and the quality of evidence. Authors can rely on several types of evidence, such as personal experiences, expert judgments, examples, statistical data, and research (Jacobsen et al., 2018). After identifying the type of evidence, students can think of how the evidence was produced and whether this process is reliable or not (Chinn et al., 2014).

Nussbaum’s (2020) guiding questions also ask students to consider other claims and conclusions that would fit the evidence, and whether something important is missing. Finally, when evaluating controversial scientific arguments, an additional point to consider is values: Are reasons supporting one side more important than reasons supporting the other side? The answers to these guiding questions allow students to make a reasonable assessment of the overall strength and plausibility of the argument.

To conclude, when making decisions on what to believe, students should not rely on only one credibility aspect but, rather, use multiple credibility aspects (Forzani, 2020). By relying on only one credibility aspect, students may, for example, reject research-based information by an expert simply because the text is published on a blogging site. Under certain circumstances, a blog might be less credible; if published by an expert, however, the information could be very important.

Asking students to think aloud while reading online or providing them with evaluation prompts are common methods to examine students’ skills as they evaluate online information. Think-aloud methodology has been used to access readers’ spontaneous evaluation of online information (Cho et al., 2018; Gottschling et al., 2020; Walraven et al., 2009). Even though think-aloud studies have shown that many students seldom evaluate online information, they have also shed light on readers’ evaluation criteria, as well as on the practices of competent and less competent readers.

Other studies have scaffolded students’ credibility evaluations with written prompts (e.g., Coiro et al., 2015; Hämäläinen et al., 2020) or representational tools (Barzilai et al., 2020; Kiili et al., 2019). While prompts may support readers’ evaluation of online information (Gerjets et al., 2011; Kammerer et al., 2016), these studies have offered additional information about readers’ capacities to evaluate online texts. Studies that have used general prompts asking readers to justify their credibility ratings or page rankings (Hämäläinen et al., 2020; Kiili et al., 2019; Mason et al., 2014) have elucidated the extent to which readers attend to different aspects of credibility.

Coiro et al. (2015) used more specific prompts when asking 773 middle school students to evaluate and justify author expertise, authors’ point of view, and overall credibility of online texts. The study showed that many students provided unacceptable or superficial criteria for their evaluations. The current study continues this line of research by asking students to rate four credibility aspects, that is, author expertise, author benevolence, publication venue, and quality of evidence, in four online texts that varied in quality.

## Individual Differences in Credibility Evaluation

Even though adolescents may be aware of the need to evaluate online information, their skills may vary in sophistication (Bråten, Brante, et al., 2018; Bråten, Stadtler, et al., 2018; Freeman et al., 2020), as may their commitment to invest the effort required to evaluate the information they find online (List & Alexander, 2018; Paul et al., 2017). Previous research has reported inter-individual differences in students’ evaluations of source expertise (Coiro et al., 2015; Kammerer et al., 2021), benevolence (Kiili et al., 2018; Potocki et al., 2020), and quality of evidence (Hämäläinen et al., 2021; Jacobsen et al., 2018). Several cognitive factors, such as prior knowledge and attitudes (e.g., Bråten et al., 2011; van Strien et al., 2016), and motivational and affective factors, such as self-efficacy and emotions (Andreassen & Bråten, 2013; Martel et al., 2020), may explain these differences.

Reading comprehension models and empirical evidence indicate that readers’ prior knowledge supports reading comprehension (Cervetti & Wright, 2020; McCarthy & McNamara, 2021). Given the fundamental role of prior knowledge in meaning making with texts, it can be expected that students with more prior knowledge would also be better able to evaluate the credibility of texts than would students with less relevant background knowledge (Afflerbach et al., 2014). The facilitative role of prior knowledge has been shown in several studies comparing credibility evaluations of domain experts and novices (Brand-Gruwel et al., 2017; Lucassen & Schraagen, 2011; Wineburg, 1991). For example, a think-aloud study by Brand-Gruwel et al. (2017) found that when reading online, domain experts relied on more specific credibility judgments than did domain novices, who tended to rely more on superficial judgments. The differences in the evaluation criteria resulted in experts selecting more credible sources than novices. A facilitative role of prior topic knowledge in evaluating texts has also been found among students across different educational levels (e.g., Bråten et al., 2011; Forzani, 2018).

Another factor that has been studied in relation to credibility evaluations and judgments is readers’ epistemic beliefs (e.g., Barzilai & Eshet-Alkalai, 2015; Barzilai et al., 2015; Kammerer et al., 2021; Wiley et al., 2020), which refer beliefs about the nature of knowledge and the process of knowing (Hofer & Pintrich, 1997). To understand epistemic beliefs concerning the justification of knowledge claims encountered on the Internet, in particular, Bråten, Brandmo, et al. (2019) developed the Internet-Specific Epistemic Justification Inventory. The inventory can shed light on the extent to which online readers believe that knowledge found on the Internet should be justified in terms of their own prior knowledge and reasoning (i.e., personal justification), the expertise of the source (i.e., justification by authority), and corroboration of information by checking multiple sources (justification by multiple sources).

A think-aloud study by Kammerer et al. (2021) found that undergraduates’ beliefs in justification by authority positively predicted comments regarding source evaluation while reading search results and websites on a controversial health issue. Another study (Hämäläinen et al., 2021), including more than three hundred upper secondary school students, examined associations between Internet-specific justification beliefs and students’ evaluation performance when using online information to solve a problem concerning either vaccines or saturated fats. Across both topics, students who believed that knowledge claims should be justified by authority or multiple sources performed better when asked to evaluate the credibility of self-selected online texts.

Recently, it has been argued that more attention also should be paid to motivational and affective factors associated with students’ evaluation of information (List & Alexander, 2018). List and Alexander (2018) reported observations suggesting that students who feel disengaged in school tasks may be content with the minimum requirements of the task and rarely evaluate information deliberatively. In line with these observations, Bråten, Brante, et al. (2018) 2018 found that behavioral engagement was associated with students’ success in a multiple document literacy task. In this task, upper secondary school students were asked to select texts and justify their selections to compose a letter to an editor on a socio-scientific topic. Behavioral engagement, measured by time on the selection task, number of texts accessed, and length of the written justifications for text selections, contributed to the number of content-based and source-based justifications for text selections even after controlling for reading comprehension and topic interest. Behavioral engagement may matter, in particular, when readers complete challenging literacy tasks, such as the students encountered in the present study (Goldhammer et al., 2014).

## Research Questions

We examined students’ abilities to evaluate the credibility of four online texts that concerned the effects of sugar consumption. In particular, we were interested in how well students evaluated four different credibility aspects of online texts that, by design, varied in quality. We also examined the potential contributions of prior topic knowledge, Internet-specific epistemic justifications, and behavioral engagement to the quality of students’ justifications for their credibility evaluations. Based on previous research, we expected students’ prior topic knowledge (Bråten et al., 2011; Forzani, 2018), Internet-specific epistemic justification beliefs (e.g., Hämäläinen et al., 2021; Kammerer et al., 2021), and behavioral engagement (Bråten, Brante, et al., 2018; Goldhammer et al., 2014) to be positively associated with credibility justifications. Specifically, we addressed the following research questions:

1. How do the participating upper secondary school students evaluate online texts that vary in quality in terms of the credibility aspects of author expertise, benevolence of the source, venue publication practices, and the quality of evidence?
2. How well do participants justify their evaluations?
3. How are participants’ prior topic knowledge, Internet-specific epistemic justification beliefs, and behavioral engagement associated with students’ credibility justifications?

## Methods

### Participants

Participants were 73 upper secondary school students, aged 16 to 17 years from two schools located in two major cities in Finland. The grade point averages required to enter these schools was considerably high. Specifically, in 2020, the required grade point averages for admission to the participating schools were 8.25 and 8.75, respectively, with the required averages across Finland varying from 5.92 to 9.85 (Vipunen—Education Statistics Finland, n.d.). After comprehensive school, students can apply either to general upper secondary school or vocational school, and in general upper secondary school, females are over-represented. In 2020, 58.5% of students completing upper-secondary school were females. In this study, 68.5% of the students were females, 28.8% were males, and 2.7% of the students abstained from assigning themselves according to the traditional gender binary. Most of the students (90.4%) indicated that they spoke Finnish at home. The remaining students (9.6%) indicated that they were bilingual and spoke Finnish and some other language at home.

### Measures

#### Prior Topic Knowledge

To assess students’ prior knowledge of sugar, we developed a topic knowledge test with 12 true–false items. The items addressed factual knowledge of sugar and its health effects. A preliminary version of the test was reviewed by a former health science teacher and a Ph.D. in medicine. The review process resulted in small modifications of some expressions and the replacement of two slightly ambiguous items. The preliminary version was piloted with 29 upper secondary students. The piloting revealed that some items were too easy, including three items with no variance. As a result, we replaced two items and modified the expressions of three items. The Ph.D. in medicine reviewed the final items. No changes were needed in this review round.

The final prior topic knowledge test included items such as “Vegetables do not have any sugar” and “Substantial sugar intake decreases blood insulin levels.” Four items had little or no variance and were therefore excluded. Hence, the maximum score on the *prior topic knowledge* measure was eight points. The score reliability was estimated using a latent variable modeling approach suitable for binary items (Raykov et al., 2010). The reliability was 0.81.

#### Internet-Specific Epistemic Justification Inventory

Students’ beliefs about justification for knowing on the Internet were assessed with the Internet-Specific Epistemic Justification Inventory (ISEJ), which has been validated with Norwegian pre-service teachers (Bråten, Brandmo, et al., 2019,2019), German university undergraduates (Kammerer et al., 2021), and Taiwanese upper secondary school students (Cheng et al., 2021). This measure targets students’ epistemic beliefs concerning the justification of knowledge claims encountered on the Internet. The measure was adapted by translating it to Finnish and contextualizing the items in terms of general school tasks. The Finnish adaptation of this measure has been shown to validly measure Internet-specific epistemic justification beliefs among Finnish upper secondary school students (Hämäläinen et al., 2021).

Students were asked to rate their justifications for knowing when using the Internet as a knowledge resource for school tasks by means of 12 items. These items represented the following three dimensions*: Personal Justification* (e.g.,”When I read a text related to my task on the Internet, I evaluate whether the information corresponds to what I already know”), *Justification by Authority* (e.g.,”When I read information related to my task on the Internet, I evaluate whether the text is written by an expert”), and *Justification by Multiple Sources* (e.g.,”To find out whether information I find on the Internet is plausible, I compare multiple information sources”). A 7-point Likert-scale was used (Describes me: 1 = very poorly, 2 = poorly, 3 = quite poorly, 4 = not poorly nor well, 5 = quite well, 6 = well, 7 = very well). Reliability was assessed with McDonald’s omega, yielding estimates of 0.74 for Personal Justification, 0.86 for Justification by Authority, and 0.93 for Justification by Multiple Sources.

The dimensionality of the ISEJ scores was examined using a principal factor analysis with oblique rotation. The item “I evaluate whether information found on the Internet appears to be logical” did not load on its designated factor (Personal Justification) but on the factor Justification by Multiple Sources (loading = 0.370) to which it was moved. All other items loaded on their designated factors. The other loadings in Justification by Multiple Sources ranged from 0.770 to 0.955. The loadings on the Justification by Authority factor ranged from 0.652 to 0.935, and the loadings on the Personal Justification factor from 0.464 to 0.939. Taken together, the factors explained 71% of the variance in the data. Based on these results, we created three mean score variables: *Justification by Multiple Sources* (five items), *Justification by Authority* (four items), and *Personal Justification* (three items).

#### Critical Online Reading Assessment (CORA)

In this study, students completed a task in a web-based environment called the Critical Online Reading Assessment (CORA). The CORA is designed to measure students’ abilities to analyze and evaluate online texts that vary in quality. The CORA recorded students’ responses, actions, and time stamps.

##### Text materials

In completing the task, students read four online texts about the effects of sugar consumption. To increase the authenticity of the texts, we designed the four fictitious texts around two health myths that spread on the Web: 1) sugar causes hyperactivity in children, and 2) sugar boosts memory. When designing the texts, we manipulated purported text authorship to present texts that would vary along the four credibility criteria, including author expertise and benevolence (see Table 1).

**Table 1:** Summary of the Texts

Text title	Description	Author	Intention	Venue	Main claim	Evidence	Words
Why not have sugar-free birthday parties?	A persuasive text that appeals to parents not to offer candies at birthday parties	Mother, works at the cash desk	Persuasion	Website provided by a blog service	Sugar causes hyperactivity in children	Mother's own observation	112
Children's sugar high—true or false?	A newspaper article where a pediatrician is interviewed about whether children get "high" from sugar	Journalist, health and well-being	Informing	Newspaper website	Sugar does not cause hyperactivity in children	Expert statement	119
How can you boost your memory in exams?	A candy company encourages students to eat "memory boosting candy" before an exam in school	Chief Executive Officer (CEO)	Commercial	Company website	Sugar improves memory	Customer survey	110
How does sugar affect our memory?	A "Researchers Answer" column shares information about sugar and its effects on memory	Researcher, PhD in health sciences, specialized in human memory	Informing	Research center website	Sugar is essential for memory functions, but excessive use of sugar is harmful to memory	Research results	115

Because of the nature of the Finnish language, the number of words would be higher in English

The online text that argued that sugar causes hyperactivity was a blog text written by a mother who pleads with other parents not to offer candies at birthday parties. She uses her own observations of her daughter’s behavior after a birthday party to develop her argument. The matched online text arguing the opposite was an article by a journalist. The text was published on a newspaper website. The journalist, specialized in health and well-being, supported the view that sugar does not cause hyperactivity with a statement from a medical expert.

The online text claiming that sugar improves memory was written by a chief executive officer of a candy company and published on the company’s website. He supported his claim with an example from a customer survey. On the contrary, a researcher and a doctor of health sciences specialized in memory research showed that sugar might have both positive and negative effects on memory. To support both sides, the researcher used two studies, both referenced explicitly at the end of the online text.

All four online texts were similar in length (range 110–119 words), number of sentences (range 13–14 sentences), and number of words per sentence (range 8.0–8.9 words per sentence). In addition, the titles were presented in the form of a question. All texts contained three paragraphs, and the main claim was presented at the end of the first paragraph. To increase the authenticity, the online texts included some pictures, and a graphic designer created logos for the company and research center websites. Students were informed that the texts were fictitious and designed for this task. Students were, however, asked to evaluate the online texts as they would evaluate similar authentic websites.

##### Task

Students’ task was to read four online texts and order them according to their credibility. While reading the texts, students responded to questions representing three types of items: *Identification items* asked students to identify the author, publication venue, main claim, and evidence. *Evaluation items* asked students to evaluate author expertise (Evaluate how much expertise the author has about the effects of the sugar), the benevolence of the author (Evaluate how committed the author is to share accurate information), the venue’s publication practices (Evaluate how well the publication venue can ensure that information on the website is accurate), the quality of evidence (Evaluate how well the author can support his/her main claim), and the overall credibility. The rating scale used ranged from 1 to 6 (e.g., 1 = very little/not very well; 6 = very much/very well). *Justification items* asked students to write a justification for their evaluations of author expertise, author benevolence, publication practices, and quality of evidence, respectively.

**Fig. 1:**
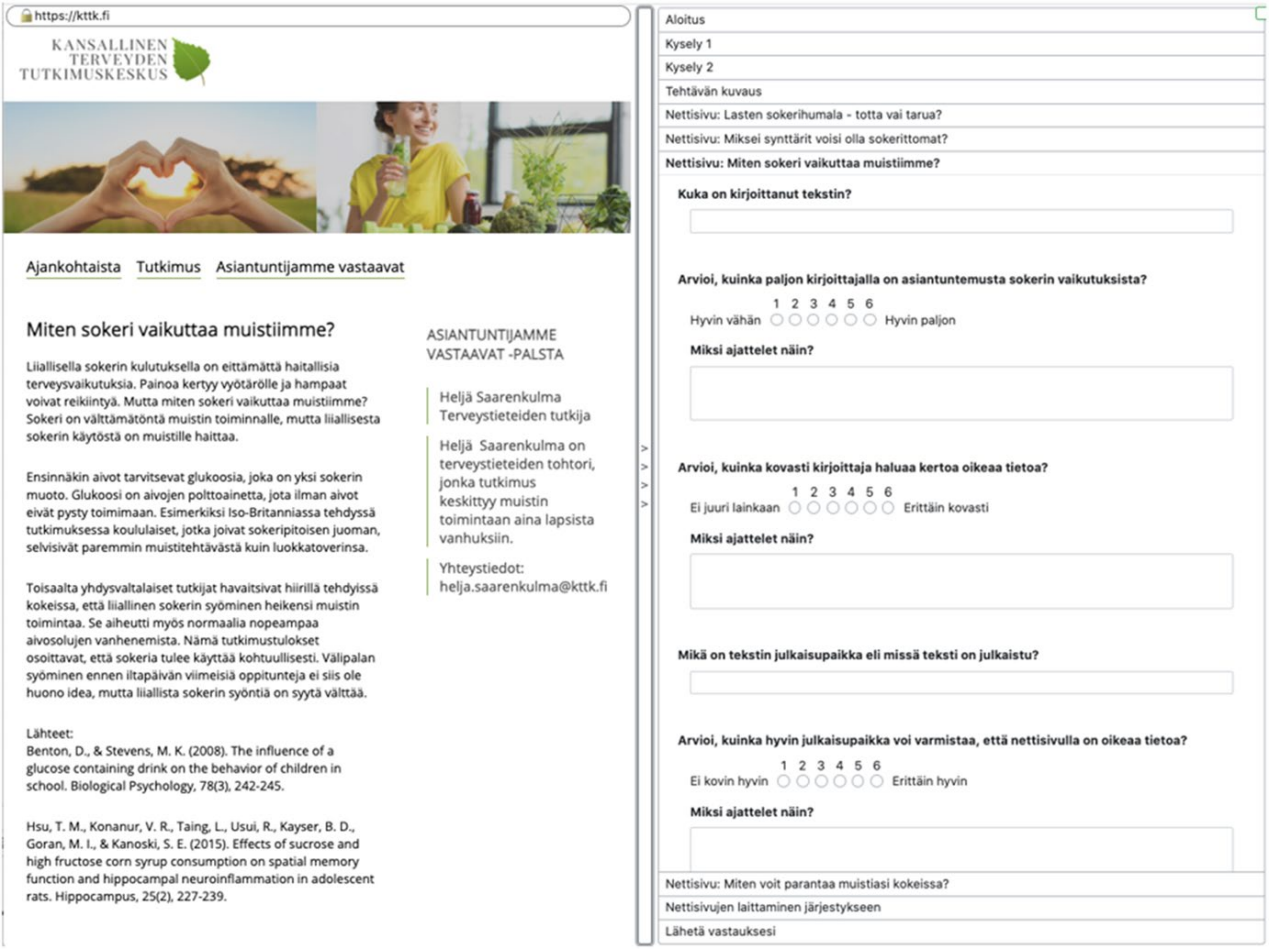
Screenshot of The CORA. On The Left-Hand Side Is The Online Text Of The Research Center, And On The Right-Hand Side Are The Questions That Students Responded To. See Appendix For An English Translation Of The Text

The task interface was divided into two parts (see Fig. 1). On the left-hand side, students saw the online text, and on the right-hand side, the instructions and the questions. After reading all four online texts and answering the questions about them, students were asked to order the text according to their credibility. The ranking task was shown on the right-hand side of the interface, and on the left-hand side, students could browse their responses to all items.

##### Behavioral engagement

Students’ behavioral engagement was measured by recording the time (in seconds) that students spent on the task.

##### Credibility Justifications

During the task, all students were asked to write 16 justifications (*N* = 1168), one for each credibility evaluation. The mean length of the justifications was 13.76 words (*SD* = 10.00). We used qualitative content analysis (White & Marsh, 2006) to examine students’ justifications for their credibility evaluations. We used both deductive procedures relying on theoretical views of sourcing (e.g., Bråten, Stadtler et al., 2018) and quality of evidence (e.g., Hoeken, 2001; Nussbaum, 2020), and inductive procedures to reflect the observed data (Bogdan & Biklen, 2003).

The analysis proceeded in two phases. The first phase aimed at creating a scoring system that could be applied to all responses representing the different aspects of credibility across the four online texts. This phase resulted in a scoring rubric with four levels (Levels 0 to 3) based on two criteria. First, we considered the relevance of the justifications in relation to the prompted credibility aspect and the properties of the evaluated text. Second, at the two highest levels, we also considered the number of justifications or the depth of reasoning.

At the highest level (Level 3), justifications had to be *elaborated and relevant.* At this level, at least two relevant justifications or a justification that was warranted, explained, or combined with a rebuttal had to be provided for a credibility evaluation. To be scored at Level 2, *one relevant justification* was required for an evaluation. At Level 1, justifications were tangential or vague. A *tangential or vague justification* supported the general direction of the evaluation but leaned on other aspects of credibility than the aspect mentioned in the prompt. This means, for example, that an evaluation of the author’s expertise could be justified by referring to the content of the text (e.g., evidence presented by the author). An *inadequate justification* (Level 0) was unclear, irrelevant, verbalized the same information that was included in the identification or evaluation task, or included a misunderstanding.

In the second phase of the analysis, we examined students’ responses concerning each credibility aspect at a time to identify representative examples of the four levels of credibility justification. Tables 2, 3, 4 and 5 include examples of the four levels for each credibility aspect. The authentic examples in Finnish have been translated into English.

**Table 2:** Analysis of Students’ Justifications for their Evaluations of Author Expertise

Quality of justification	Mother’s blog	Newspaper's website	Company website	Research center's website
**Level 3:** Elaborated, relevant justification (3 points)	*"She is a mother of three children, and she does not tell anything about her education"* (Student 2LT)	*"The author is a journalist, who is specialized in health and well-being, suggesting that he has acquainted himself with the topics that he deals with" *(Student 2V5)	*"The author is a CEO. He is not an expert in sugar effects or, for example, an expert in health issues" *(Student 2SY)	"*She is a researcher in health sciences. Thus, she has research-based knowledge about the topic. She understands the biological functions of sugar. The author has particularly focused on children's memory functions*" (Student 15 M)
**Level 2:** Relevant justification (2 points)	*"She does not have education and only relies on observations *(Student 139)	*"After the name of the author, it is added "health and well-being", so he might have some knowledge about the issue or at least some interest *(Student 3FP)	*"Because he is not an expert in health issues"* (Student 175)	*"She is a doctor in health sciences"* (Student 1IJ)
**Level 1:** Tangential or vague justification (1 point)	*"She does not have any research evidence. The claim is based on her own experiences" *(Student 3JT)	*"Author reports other person's observations, such as statements by the doctor of the children’s hospital, Market Valtasalo"* (Students 3E7)	*"He just wants to advertise his candy store"* (Student 10G)	*"The text presents factual knowledge that has been supported with references"* (Student 3Z1)
**Level 0:** Inadequate justification (0 points)	*"Because she only justified why her children go wild"* (Student 2HU)	*"He is a doctor who has investigated the issue"* (Student 3AR)	*"Author has some basic knowledge, but he presents it in an unusual way"* (Student 3NS)	*"Because she has clearly taken most of the text from different sources, so this is not her own knowledge about sugar and its effects"* (Student 3BQ)

**Table 3:** Analysis of Students’ Justifications for their Evaluations of Author Benevolence

Quality of justification	Mother's blog	Newspaper's website	Company website	Research center's website
**Level 3: **Elaborated, relevant justification (3 points)	*"The author does not get any benefits from delivering this particular information. The author has own children, so she probably feels obligated to deliver information that she thinks is useful"* (Student 24X)	*"As the author is a journalist, he wants to stick to the truth because delivering false information would have negative consequences for his reputation. In addition, there are no commercials on the page, so he does not have a commercial motive to do a click article"* (Student 2V5)	*"The author wants to increase the sales, so he does not talk negatively about sugar effects, even if it has negative effects. Of course, if the information is false, the company might be in trouble. Thus, the author tries to avoid that"* (Student 1MY)	*"The author has no reason to tell inaccurate information because her job is based on delivering objective information and analysis. The article does not mention any brands, so the benefits are out of the question"* (Student 24X)
**Level 2:** Relevant justification (2 points)	*"She wants to warn/instruct other parents"* (Student 39H)	*"He wants to refute the false belief about the sugar is causing hyperactivity"* (Student 1RI)	*"The author just wants to have more customers"* (Student 2PY)	*"The text aims at delivering information about the health issues to the citizens"* (Student 1PW)
**Level 1:** Tangential or vague justification (1 point)	*"The text is more an opinion than factual knowledge"* (Student 3PL)	*"He has included research-based information in his article and interviewed the knowledgeable pediatrician"* (Student 2N2)	*"The author benefits from his writings"* (Student 23I)	*"Because she tells about different perspectives"* (Student 3AR)
**Level 0:** Inadequate justification (0 points)	*"Because not all information is correct"* (Student 3AR)	*"The author has ended up writing an article"* (Student 3FP)	*"The author writes in an encouraging way and honestly about facts without any special claims"* (Student 3TZ)	*"It seems that she does not have a reason to lie"* (Student 1FS)

**Table 4:** Analysis of Students’ Justifications for their Evaluations of the Publication Venue

Quality of justification	Mother’s blog	Newspaper's website	Company website	Research center’s website
**Level 3:** Elaborated, relevant justification (3 points)	*"In a blog, anyone can write anything. Nobody demands to check the source"* (Student 1G4)	*"Journalists’ texts are probably reviewed before publication, but there is still a possibility of some factual errors"* (Student 2QT)	*"In Finland, customers can not be lied to about benefits or harms of products, but one can, to some extent, leave something unsaid"* (Student 3ZR)	*"The website publishes information that is written by experts, and that is probably checked multiple times"* (Student 1C1)
**Level 2:** Relevant justification (2 points)	*"I do not believe that the website provider checks published information very thoroughly*" (Student 37B)	*"One can give feedback to newspapers if there are mistakes in the content"* (Student 1PW)	*"Because the website is owned by the firm, it can publish there what it wants"* (Student 2PY)	*"Experts from different fields work in the research center"* (Student 2SY)
**Level 1:** Tangential or vague justification (1 point)	*"It is a blog of a mother, who does not base her knowledge on research*” (Student 1FS)	"*Newspapers aim at transmitting correct information"* (Student 175)	"*It is an advertisement of a store, the purpose of which is to promote sales"* (Student 15 M)	*“References are marked correctly and author contact information”* (Student 2V5)
**Level 0:** Inadequate justification (0 points)	*"It is a blog"* (Student 2HU)	*“He told about the issue in a reasonable manner”* (Student 2QJ)	*"It is not correct information" (Student 3AR)*	*“Because the website is about this particular topic”* (Student 3SM)

**Table 5:** Analysis of Students’ Justifications for their Evaluations of Provided Evidence

Quality of justification	Mother’s blog	Newspaper website	Company website	Research center’s website
**Level 3:** Elaborated, relevant justification (3 points)	*"Author justifies her claim with observations done after the birthday parties without knowing about the exact events and other potential factors that may have affected her daughter's behavior"* (Student 222)	*"He has used both expert statement and research knowledge. He has acquainted himself with the issue by asking an expert to tell him about the topic and by finding research knowledge that supports his claim. However, the place of the study is not told which decreases its credibility"* (Student 3ZR)	*"The results of the questionnaire are favorable to the company. If the results would not have been favorable, the article would probably not have been published. One needs to keep in mind that participants were users of the products, and thus, biased"* (Student 222)	*"She has told a little bit about the studies. She has also told what are the exact studies in question so that one can go to check them"* (Student 2CA)
**Level 2:** Relevant justification (2 points)	*"One cannot be sure if the sugar has caused hyperactivity. One could assume that having fun with friends would get anyone excited"* (Student 1ZV)	*"The person interviewed was a doctor who is an expert"* (Student 1HL)	*"Evidence is weak, only some people's personal experiences"* (Student 1C1)	*"Because there is more than one research source"* (Student 198)
**Level 1:** Tangential or vague justification (1 point)	*"She does not have any research-based knowledge"* (Student 2TK)	*"He has a credible source. There could be additional sources"* (Student 15 M)	*"He does not use proper research"* (Student 1W1)	*"She is a professional who has several types of knowledge, justifications, and perspectives"* (Student 3J3)
**Level 0:** Inadequate justification (0 points)	*"She witnessed the situation with her own eyes"* (Student 2X8)	*"He did a study about the issue"* (Student 3LW)	*"Surveys make it plau sible"* (Student 2WB)	*"She explains ideas clearly"* (Student 2QJ)

The inter-rater reliability of the analysis of students’ credibility justifications was examined by having two persons independently score 25% of the responses. The Kappa values ranged from 0.64 to 0.93 (author expertise: 0.64–0.93; author benevolence: 0.76–0.93; publication venue: 0.77–0.92; quality of evidence: 0.64–0.84). All disagreements were negotiated, and the first author scored the remaining responses in alignment with the negotiated definitions and scoring criteria.

We created a sum variable for *Credibility Justifications* by combining students’ justification scores for author expertise, author benevolence, publication venue, and quality of evidence (16 items). In this way, we aimed to capture students’ ability to justify their credibility evaluations more broadly and more reliably instead of focusing on separate item scores. To assess the reliability of this variable, we used McDonald’s Omega, obtaining an estimate of 0.79. Two obvious outliers were removed from the analysis (one low-achieving student, who had also skipped many of the items, and one high-achieving student).

### Procedure

The study was integrated in a Finnish language arts course during the COVID-19 pandemic when teaching in upper secondary schools was implemented as distance learning. Students’ language teachers sent students an information letter about the study a few days in advance. Students were informed that they were able to do the task anonymously, and at the beginning of the task, they indicated whether their responses could be used for research purposes.

In the online class, teachers shared a Google Drive document that included a short description of the study. The document also included a link to the video that introduced the assessment environment and a link to the task environment itself. If students had not read the information letter about the study, they were given access to it. Students entered the task environment with a code, and they proceeded with the task in their own space. Before starting the actual task, students filled in their background information and answered the prior topic knowledge questionnaire and the Internet Specific Epistemic Justification questionnaire. These questionnaires were embedded in the assessment environment.

To control for possible order effects, students were randomly divided into two groups that read the texts in different orders. The students in Group A read the more credible texts first, whereas the students in Group B read the questionable texts first.

### Statistical Analysis

The statistical analyses were conducted by using SPSS 27. To examine whether the quality of students’ credibility justifications for author expertise, author benevolence, publication practices of the venue, and quality of evidence differed across the texts, we conducted four Friedman’s tests (one for each credibility aspect). The pairwise comparisons were run with the Wilcoxon rank test. The non-parametric methods were used because of the non-normality of some variables. We used the correlation coefficient *r* as effect size measure with a 95% confidence interval (CI).

To examine how prior topic knowledge, internet-specific epistemic justification beliefs, and behavioral engagement were associated with students’ credibility justifications (Research Question 3), we conducted a sequential regression analysis (Tabachnick, & Fidell, 2013). Although we were interested in the contribution of each variable or set of variables (epistemic justification beliefs), we conducted the analysis in three steps. In the first two steps, we entered the variables measured prior to the critical online reading task. Specifically, in Step 1, we entered prior topic knowledge because it can be considered a foundational component in reading comprehension. In Step 2, we entered the three internet-specific epistemic justification variables. Finally, in Step 3 we entered the variable time on task (measured in seconds) to examine whether behavioral engagement would predict students’ credibility justifications over and above their prior topic knowledge and epistemic justification beliefs.

## Results

### Credibility Evaluations

**Fig. 2:**
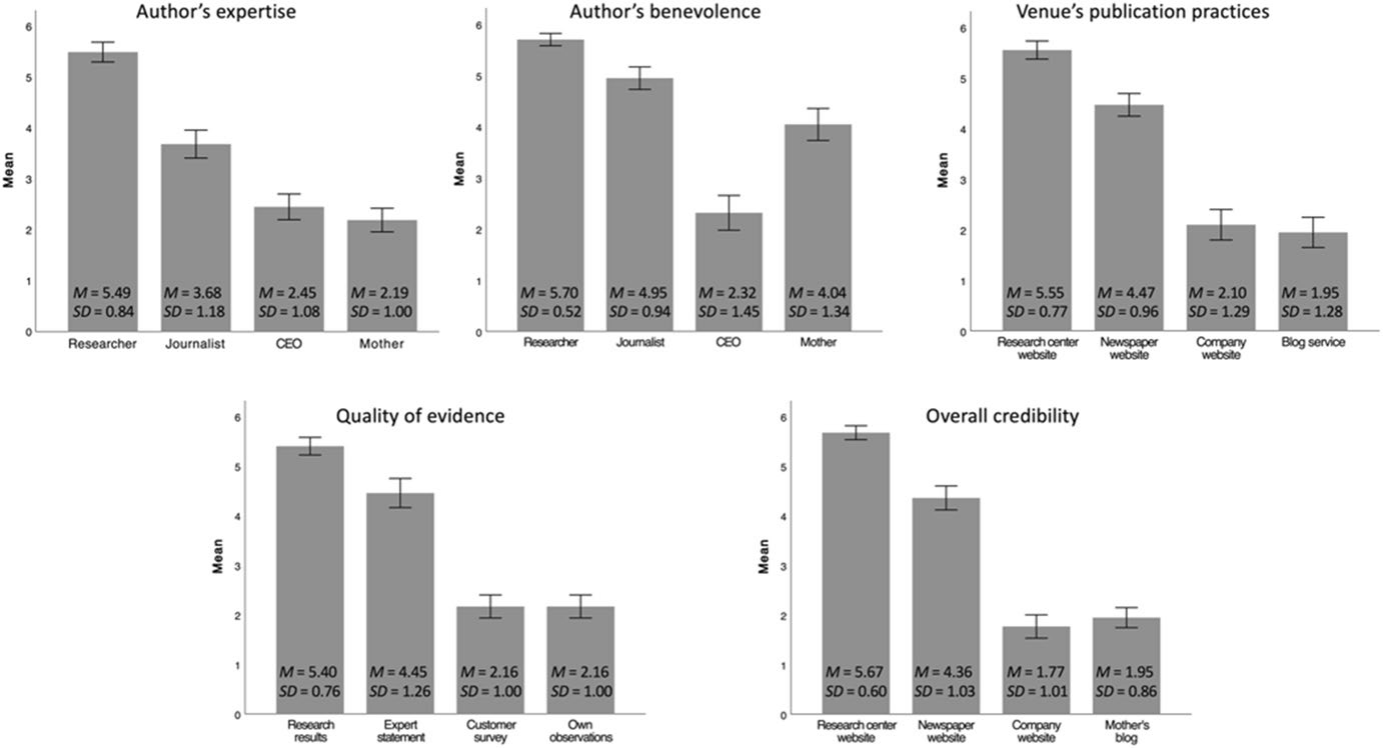
Students’ (N = 73) Evaluations of Author Expertise, Author Benevolence, Venue’s Publication Practices, Quality of Evidence, and Overall Credibility (Scale from 1 to 6, with 1 Indicating the Lowest and 6 the Highest Value). Error bars indicate 95% confidence intervals.

As shown in Fig. 2, students, with very few exceptions, were able to differentiate the expertise, benevolence, publication practices of the venue, quality of evidence, and overall credibility of the more and less credible online texts accurately. For example, students evaluated the author expertise of the researcher highest, followed by the journalist, whereas the CEO’s and the mother’s expertise were evaluated lowest. Similarly, the research evidence was rated highest, and the customer survey and mother’s observations were evaluated considerably lower. Notably, 13.7% of the students rated the benevolence of the CEO as high without questioning his intentions.

### Credibility Justifications

Table 6 presents descriptive information about students’ justifications for their evaluations by credibility aspect. Students’ justifications, with some exceptions, were not very sophisticated. To the contrary, many justifications were merely tangential, suggesting that students’ justifications were in line with their evaluations but did not focus on the specific credibility aspect that was targeted. For example, when students were asked to justify their expertise evaluations, many focused on the text content. One explanation for this finding might be that students regarded text content as evidence of the author’s knowledge base.

**Table 6:** Descriptive Statistics for Students’ Credibility Justifications by Credibility Aspect (N = 69–71)

Credibility aspect	*M*	*SD*	*Md*	Pairwise comparisons
***Author expertise***				
1. Researcher	1.82	0.97	2	1 > 2, 3, 4
2. CEO	1.36	0.89	1	
3. Mother	1.31	0.62	1	
4. Journalist	1.07	0.95	1	
***Author benevolence***				
1. CEO	1.96	0.78	2	1 > 2, 3,4
2. Mother	1.39	0.87	1	
3. Researcher	1.38	0.73	1	
4. Journalist	1.09	0.85	1	
***Venue’s publication practices***				
1. Blog service	1.41	0.94	1	1 > 4
2. Research center	1.11	0.99	1	
3. Newspaper	1.06	1.06	1	
4. Company	0.96	0.73	1	
***Quality of evidence***				
1. Own observations (Blog)	1.18	0.95	1	
2. Expert statement (Newspaper)	1.17	1.17	1	
3. Customer survey (Company)	1.14	1.07	1	
4. Research results (Research center)	1.10	0.97	1	

Students’ justifications for their expertise evaluations differed across the online texts (*χ*^*2*^(3) = 32.22, *p* < 0.001). Post-hoc comparisons showed that students performed better in justifying the researcher’s expertise than in justifying any other author’s expertise. The effect sizes (*r*) varied from 0.44 to 0.55 indicating medium to large effect sizes (Researcher vs. CEO: *r* = 0.44; 95% confidence interval *CI* = [0.23,-0.62]; Researcher vs. Mother: *r* = 0.43; *CI* = [0.22-0.62]; Researcher vs. Journalist: *r* = 0.55; *CI* = [0.36-0.70]). This seems reasonable, in part because the research center website also provided more information about the author than the other websites. For the mother, journalist, and CEO, students may have needed to make more inferences about their expertise. Students scored lowest when justifying their evaluations about the journalist’s expertise, and only 25% of the students presented one or more relevant justifications (score 1 or 2). Even though all students, with one exception, were able to identify the correct author (journalist), almost 10% of the students evaluated the doctor who was interviewed in the text instead of the journalist. Interestingly, two students thought that the researcher did not have knowledge because she only referred to research done by others.

With respect to author benevolence, students’ performance also differed across the texts (*χ*^*2*^(3) = 49.43; *p* < 0.001). Judgments of the CEO’s benevolence were generally better justified than the benevolence of the other authors. The effect size (*r*) for CEO vs. Mother was 0.45, 95% CI = [0.24-0.62], for CEO vs. Researcher 0.57, CI = [0.39-0.71], and for CEO vs. Journalist 0.68, CI = [0.53- 0.79]. One potential explanation for this might be that the commercial intentions were rather obvious on the website. In contrast, when justifying the benevolence of the journalist or the researcher, the text provided no explicit hints about their benevolent intentions, which meant that students needed to rely on their prior source knowledge for their justifications.

Similarly, there were differences across the texts in how well students justified their evaluations concerning the publication practices of the venue (*χ*^*2*^(3) = 13.18; *p* = 0.004). The post-hoc comparisons showed that students’ justifications concerning the blog service publication practices were of higher quality than were those for the company’s website (*r* = 0.46, 95% CI = [0.25-0.63]. Even though students scored highest in justifying their evaluations for the publication practices of the blog service, some students’ answers reflected the idea that blogs are reserved for personal opinions only. Only one student referred to the journalist guidelines when justifying her evaluation of the publication practices of the newspaper. This student even mentioned the Council of Mass Media (see https://www.jsn.fi/en/), who has published the guidelines.

As Table 6 shows, the means of the credibility justifications for the quality of evidence were low across the online texts. Only 38.3% of the students were able to present at least one relevant justification for their evaluation of the mother’s observation. For example, 23% of the students considered other potential factors than sugar as a cause of the children’s hyperactivity at the birthday party. The proportions of relevant justification(s) were 41% for the customer survey, 46.5% for the expert statement, and 47.2% for research.

Descriptive statistics for all variables included in the regression analysis, as well as their inter-correlations, are presented in Table 7. Students did not score particularly high in overall credibility justification, obtaining a mean score of 20.21 out of 48. In addition, the variation in students’ scores was considerable, with a standard deviation of 7 points.

**Table 7:** Descriptive Statistics and Inter-Correlations for All Variables Included in the Regression Analysis (N = 71)

Variable (max score)	1	2	3	4	5	6
1. Credibility justifications (48)		.249^*^	.273^*^	.236^*^	.108	.356^**^
2. Prior topic knowledge (8)			.297^**^	.165	.051	.315^**^
3. Justification by multiple sources (7)				.476^***^	.470^***^	.208
4. Justification by authority (7)					.287^**^	.069
5. Personal justification (7)						.014
6. Time spent on task						
*M*	20.21	6.17	5.42	4.63	5.32	38:48
*SD*	7.00	1.19	1.04	1.17	0.90	12:29
Skewness	-0.22	-0.12	-0.65	-0.43	-0.76	0.59
Kurtosis	-0.55	-0.49	0.20	-0.34	1.11	0.49

^***^p < .001, ^**^p < .01, ^*^p < .05

Table 8 presents the results of the regression analysis predicting students’ performance with respect to overall credibility justification. In Step 1, prior topic knowledge was a statistically significant predictor explaining 6.2% of the variance in students’ performance, *F*(1, 69) = 4.572, *p* = 0.036. In Step 2, with the three Internet-specific justification belief variables included in the equation, the model was only marginally statistically significant, *F*(4, 66) = 2.234, *p* = 0.075. In Step 3, with time on task also included in the equation, the model was statistically significant, *F*(5, 65) = 3.122*, p* = 0.014, explaining 19.4% of the variance of credibility justification. Only time on task was a statistically significant predictor in this model, indicating that the more time students spent on the task, the higher the quality of their credibility justifications was.

**Table 8:** Results of Sequential Regression Analysis for Variables Predicting Students’ Overall Credibility Justification (N = 71)

Variable		*β*	*R*^*2*^	*ΔR*^*2*^
Step 1			.06^*^	
	Prior topic knowledge	.25^*^		
Step 2			.12	.06
	Prior topic knowledge	.18		
	Personal justification	-.02		
	Justification by authority	.13		
	Justification by multiple sources	.16		
Step 3			.19^*^	.07^*^
	Prior topic knowledge	.10		
	Personal justification	-.00		
	Justification by authority	.15		
	Justification by multiple sources	.11		
	Time on task	.29^*^		

^*^p < .05

## Discussion

The goal of the present study was to gain further understanding of how students evaluate different credibility aspects of online texts that differ in quality and how individual differences may contribute to their justifications for those evaluations. To attain this goal, we manipulated four online texts with respect to the author’s expertise, the author’s intention, the publication practices of the venue, and the quality of the evidence, and asked students to evaluate each of these aspects on a six-point scale and justify their evaluations. In addition, we examined the role of prior topic knowledge, Internet-specific epistemic beliefs, and time on task in students’ justifications of their credibility evaluations.

### Students Were Skilled in Differentiating More Credible From Less Credible Texts

In general, students were able to evaluate the different credibility aspects and overall credibility of online texts quite accurately, which is an ability that is still developing among younger students (Kiili et al., 2018). Still, there were some students (13.7%) who struggled to question the author’s intention regarding the commercial online text, even though the commercial nature of the text was obvious. In conclusion, however, it seemed that the textual materials used in the assessment task were quite easy for most upper secondary school students to evaluate, given that all credibility aspects were accurately rated by the majority of participants as being of either higher or lower credibility overall. For example, the commercial text included clear hints, such as advertising slogans, about the potential commercial bias. For students at this age, future studies could examine credibility evaluation using more challenging textual materials (cf., McGrew et al., 2018). For example, the textual materials could include a text by an interest organization representing producers or sellers of certain products. The questioning of the credibility of sources with more hidden agendas would sometimes require an understanding of underlying mechanisms of lobbying, which is, presumably, quite challenging even at this educational level.

### Many Students Struggled With Justifying Their Credibility Evaluations

Despite being capable of differentiating the more credible texts from the less credible texts, many students’ credibility justifications were not particularly sophisticated, with students scoring 20 points out of 48 on average. This finding is in line with previous studies (e.g., Coiro et al., 2015; McGrew et al., 2018). On some occasions, students focused on other aspects of credibility than what was asked for, provided vague justifications, or sometimes even provided irrelevant information. For example, when asked to evaluate the author’s expertise, some students justified their credibility evaluations by referring to text content instead of evaluating the source of the text, and vice versa.

Interestingly, students struggled particularly with justifying the credibility evaluation of the journalistic online text across all credibility aspects. One possible reason is that students are relatively unaware of journalistic processes and norms (Karlsson, 2011), or of journalists’ expertise as professional information seekers or expert generalists (see Kohnen & Mertens, 2019). For example, some students, very few though, had a naïve belief that journalists do not have any knowledge about the topic they are writing about before interviewing an expert. However, it might also be the case that the fast continuous news cycle and user participation in online news production have altered the authority associated with journalism (Karlsson, 2011). An additional, potentially confounding factor that should be considered when interpreting our findings is that we used a fictitious newspaper as a source. The results might have been different if we had used an authentic, well-established news source.

Further, it seemed that the process of justifying the quality of evidence was difficult for many participants. For example, even though students were considering research high-quality evidence supporting the main claim of the author, a little less than half of the students (47%) were able to present at least one relevant justification for their evaluation. Students may have learned to consider research as something to trust without being able to articulate, for example, why research processes can be regarded as reliable ( Chinn et al., 2014). That said, some students were able to think scientifically when justifying why one should discount the mother’s observation as reliable evidence, with these justifications, for example, including consideration of alternative explanations and lack of generalizability.

One explanation for the observation that participating students struggled to justify their evaluations of the quality of evidence may relate to the way evaluation of online texts has been taught in school. Instruction may have focused on the evaluation of sources and source features instead of the quality of evidence. Focus on sourcing is also a prevalent trend in intervention studies promoting credibility evaluations of multiple documents (Bråten, Brante, et al., 2019; Pérez et al., 2018). On the other hand, instruction related to argumentation may have focused on teaching students to create strong and effective arguments with less focus on evaluating arguments in terms of the quality of evidence (cf. Cartiff et al., 2021). Future research should therefore take what students have been taught about the evaluation of Internet texts into account.

### Individual Differences: Time on Task Mattered the Most

There were considerable inter-individual differences in students’ performance when justifying the credibility evaluations. Students’ credibility justification score ranged from 3 to 42 out of 48, with a standard deviation of 7. Previous studies have also observed similar, substantial differences among students’ abilities to judge the credibility of online texts (e.g., Coiro et al., 2015; Forzani, 2018; Kiili et al., 2019).

In the present study, we examined whether prior topic knowledge, Internet-specific epistemic justification beliefs, and behavioral engagement (i.e., time on task) could explain some of these differences. Our results showed that prior topic knowledge explained 6% of the variance in students' performance with respect to credibility justifications. Although prior topic knowledge has been shown to have a relatively large effect on reading comprehension (McCarthy, & McNamara, 2021), its contribution to credibility justifications seemed to be considerably smaller, which is consistent with previous studies among adolescents (Forzani, 2018; Hämäläinen et al., 2021). Presumably, there are other types of prior knowledge, such as knowledge about sources, genres, or evidence types, that may play a role in credibility judgments beyond prior knowledge about the content of the texts.

Further, we were interested in the role of Internet-specific justification beliefs in the quality of students’ credibility judgments. Inconsistent with previous findings (Hämäläinen et al., 2021; Kammerer et al., 2021), the internet-specific justification beliefs were only marginally significantly associated with students’ actual credibility justifications. This might be because of the small sample size of the current study.

Overall, when including all the variables of interest, they explained 19% of the variance, with time on the task being the only statistically significant independent variable. The importance of behavioral engagement has also been shown in previous studies when students are completing challenging literacy tasks (e.g., Bråten, Brante, et al., 2018; 2018 Goldhammer et al., 2014; List et al., 2019). One crucial question for all assessment developers and for those interpreting assessment results is how engagement matters, and for whom. To increase affective engagement, more attention could be put on emotional design when creating digital learning materials (Plass & Kaplan, 2016). Emotional design refers to the use of design features aiming to induce emotions that support deep processing of information. One promising direction could be design of materials that include interaction with a pedagogical agent who guides students through critical online reading tasks (Plass & Kaplan, 2016), supporting both cognitive and affective engagement.

In addition to the motivational perspective, time on task can be considered from the perspective of two types of cognitive processing that differ with respect to speed and the nature of processing (Kahneman, 2003; Stanovich & West, 2000). The faster processing is automatic, implicit, and heuristic, whereas the slower processing is effortful, analytical, and deliberative. In credibility evaluation, readers use heuristic processing when they, for example, rely on salient cues regarding reputation, persuasive intent, or expectancy violation (Metzger & Flanagin, 2013). Heuristic processing may partly explain vague, tangential, or erroneous responses in this study. For example, students relied on cues regarding persuasive intent (commercial cues) even when they were asked to judge the author's expertise. Students also relied on salient cues regarding expertise (a doctor who was interviewed) to justify the author's expertise, in this case, a journalist. Some justifications also indicated heuristics related to text genres: the blog texts include personal opinions.

### Limitations

This study has four important limitations that are worth considering when interpreting the results. First, because of the COVID-19 pandemic, students completed the assessment task during the distance teaching period at home without any supervision of a teacher or researcher. However, students responded anonymously, and the task was not high-stakes; thus, students did not have any reason to cheat. On the other hand, the possibility to respond anonymously may have caused a lack of motivation to do one’s best. To motivate students, the first author arranged a feedback online session so that students had an opportunity to relate their performance to the task expectations. Second, the results are based on a rather small sample of students who were recruited from schools that required a considerably high grade point average for entrance. Thus, one should be cautious about making any generalizations without replication in other samples and, preferably, in other cultures (cf., Putman et al., 2020).

Third, the textual materials that students were exposed to were fictitious. Students were informed about this issue and asked to evaluate the online texts as they would evaluate similar authentic texts. Despite this instruction, students may have evaluated the texts differently than authentic texts. In authentic contexts, students may have had additional source knowledge, for example, about the venue, which they could have utilized in their credibility justifications. Fourth, the assessment task included sixteen open-ended justification items. The high number of items that may have appeared quite similar to the students might have decreased some students’ motivation to respond carefully to each item.

### Instructional Implications

A critical online reader can make an informed judgment about the credibility of online texts by evaluating different credibility aspects (Forzani, 2020). Depending on the situation and task demands, a critical reader may activate different evaluation strategies from a broader toolkit. Instruction should provide students with a toolkit that allows them the flexibility to engage in deep evaluation of online information across different situations. This includes not only the ability to accurately evaluate the credibility of online texts but also the ability to justify their evaluations thoroughly. Our results suggest that adolescent readers need to be taught about criteria making texts more or less credible.

To support their students, teachers can orchestrate discussions that go beyond simple explanations of credibility that are typical for many students (e.g., references to research makes information credible). Fruitful discussions may then scaffold students’ development toward justifications that are well-reasoned, warranted, and flexible. Such discussions can include attention to sourcing, such as what constitutes relevant expertise, what kinds of intentions authors may have, and what kinds of publication practices different venues may follow.

Our study suggests that some students may have a simplistic view of expertise (e.g., that own experiences in and of themselves are plausible sources of expertise). Of course, this issue may be even more prevalent among younger students. Students could therefore benefit from analyzing the components of expertise and the various ways experts acquire knowledge. If possible, students could interview experts from different fields to learn about various ways that experts construct knowledge. After having examined expertise in this way, students could consider how to make inferences about aspects of expertise with source features provided on the websites. Finally, it would also be worth discussing the boundaries of expertise with students (Chinn et al., 2020).

Even though relying on expert knowledge is often a reliable process for determining which online texts to trust (Chinn et al., 2020), the manipulation of source information is one means of misleading readers, and, therefore, evaluation focusing solely on source information may fall short (Sinatra & Lombardi, 2020). This means that students also need to learn how to evaluate the author’s arguments, including the quality of evidence. For example, teachers could introduce different types of evidence and guide students to think about how the types of evidence were produced and how credible they are (Chinn et al., 2014). Finally, it is worth restating that becoming a strategic critical online reader requires extensive practice with various texts and contexts (Afflerbach et al., 2020).

Our results suggest that in addition to promoting cognitive aspects of credibility evaluation, teachers need to support students’ behavioral engagement. Ideally, students would have a critical analytic approach to examine multiple, contradictory online texts (List & Alexander, 2017). Students with a critical analytic approach are skilled in evaluating online resources and they are affectively engaged with materials. Finally, students would benefit from instruction that provides them with metacognitive tools that can facilitate their understanding of the demands of different situations with respect to depth of processing (Kuhn, 2021). That is, an understanding of when faster, heuristic processing might be effective and when there is a need to engage in more effortful, analytical, and deliberative processing. In brief, educators should try to ensure that adolescents can turn, whenever needed, to deliberative credibility evaluation of online information.

## Appendix

An English Translation of the Online Text presented in Figure 1.
